# Optimizing Cold Food Supply Chains for Enhanced Food Availability Under Climate Variability

**DOI:** 10.3390/foods14152725

**Published:** 2025-08-04

**Authors:** David Hernandez-Cuellar, Krystel K. Castillo-Villar, Fernando Rey Castillo-Villar

**Affiliations:** 1 Mechanical Engineering Department, Texas Sustainable Energy Research Institute, The University of Texas at San Antonio, 1 UTSA Circle, San Antonio, TX 78249, USA; david.hernandezcuellar@my.utsa.edu; 2 Facultad de Ciencias Economicas y Empresariales, Universidad Panamericana, Mexico City 03920, Mexico; frcastillo@up.edu.mx

**Keywords:** climate variability, food availability, cold food supply chain, supply chain optimization, risk mitigation

## Abstract

Produce supply chains play a critical role in ensuring fruits and vegetables reach consumers efficiently, affordably, and at optimal freshness. In recent decades, hub-and-spoke network models have emerged as valuable tools for optimizing sustainable cold food supply chains. Traditional optimization efforts typically focus on removing inefficiencies, minimizing lead times, refining inventory management, strengthening supplier relationships, and leveraging technological advancements for better visibility and control. However, the majority of models rely on deterministic approaches that overlook the inherent uncertainties of crop yields, which are further intensified by climate variability. Rising atmospheric CO_2_ concentrations, along with shifting temperature patterns and extreme weather events, have a substantial effect on crop productivity and availability. Such uncertainties can prompt distributors to seek alternative sources, increasing costs due to supply chain reconfiguration. This research introduces a stochastic hub-and-spoke network optimization model specifically designed to minimize transportation expenses by determining optimal distribution routes that explicitly account for climate variability effects on crop yields. A use case involving a cold food supply chain (CFSC) was carried out using several weather scenarios based on climate models and real soil data for California. Strawberries were selected as a representative crop, given California’s leading role in strawberry production. Simulation results show that scenarios characterized by increased rainfall during growing seasons result in increased yields, allowing distributors to reduce transportation costs by sourcing from nearby farms. Conversely, scenarios with reduced rainfall and lower yields require sourcing from more distant locations, thereby increasing transportation costs. Nonetheless, supply chain configurations may vary depending on the choice of climate models or weather prediction sources, highlighting the importance of regularly updating scenario inputs to ensure robust planning. This tool aids decision-making by planning climate-resilient supply chains, enhancing preparedness and responsiveness to future climate-related disruptions.

## 1. Introduction

Produce supply chains play a vital role in the food industry by delivering fruits and vegetables to consumers efficiently, cost-effectively, and in peak condition. However, these systems are increasingly strained by the growing impacts of climate change, one of the most urgent environmental issues of our time. Climate change introduces considerable uncertainties into agricultural productivity, driven by increased frequency and intensity of heatwaves, droughts, floods, and other extreme weather events. Fluctuations in temperature and precipitation patterns have a direct effect on crop health, leading to heightened heat stress, decreased photosynthesis rates, shorter growing periods, and diminished yields. Consequently, disruptions in produce supply chains—from farm to table—can become increasingly severe and unpredictable. Empirical research highlights the sensitivity of agriculture to climate factors; for instance, Maskey et al. [1] examined the link between diverse weather variables and strawberry yields, demonstrating robust statistical correlations. Their findings underline the necessity of incorporating climate resilience into supply chain planning to safeguard agricultural productivity and maintain consistent food availability.

Additionally, climate change introduces substantial financial burdens for farmers and stakeholders across the entire supply chain. To adapt to increasingly erratic environmental conditions, farmers may be forced to invest in advanced harvesting technologies, climate-resilient infrastructure, or irrigation systems. In extreme scenarios, relocation of agricultural operations to climatically favorable regions becomes necessary—an adjustment that entails significant increases in both production and transportation costs.

Beyond the farm level, climate-induced disruptions also impact distributors and consumers. Variability in crop yields may compel distributors to identify and contract with alternative suppliers in distant regions, thereby reconfiguring established supply chains. These adjustments often result in elevated operational costs and logistical complexity. For example, elevated temperatures and humidity levels in some regions exacerbate the difficulties of storing and transporting perishable produce, increasing the risk of spoilage and quality degradation [2]. Maintaining product quality and freshness under such conditions demands specialized infrastructure, such as cold storage and temperature-controlled logistics, further amplifying the cost and complexity of produce distribution.

This study focuses on examining a cold food supply chain (CFSC) network operated by a real-world logistics company. The company’s responsibility involves handling the storage and transportation of strawberries from farms in California to customers throughout the United States. Most of the time, the logistics company utilizes refrigerated trailers and cold storage facilities to ensure the preservation of the strawberries’ quality. To optimize the transportation cost of strawberries, a stochastic hub-and-spoke network model was developed. This model aims to identify an optimal distribution and transportation network while taking into account climate variability and its impact on crop yield. The objective is to provide the logistics company with a modern, data-driven decision support system that can assist in enhancing the CFSC in the coming years.

Strawberries are one of the most economically significant fruit crops globally, with over 9.2 million metric tons produced worldwide in 2022 [3]. The United States is among the top three producers, alongside China and Mexico. Within the U.S., California dominates production, accounting for nearly 90% of the country’s strawberry output, which totaled approximately 1.3 million metric tons in 2022 [4]. The U.S. is also a leading consumer of strawberries, with per capita consumption averaging around 3.5 kg per year [5]. This high level of consumption, paired with the crop’s perishability, makes efficient and resilient supply chains essential for maintaining product quality and controlling costs amid increasing climate variability.

### 1.1. Literature Review

Optimization models have been widely used in the design and management of supply chains, including those for fresh produce. These models can assist decision-makers in logically evaluating and planning for different possible outcomes and minimize supply chain costs while meeting demand and quality requirements. According to Nguyen et al. [2], from a modeling perspective, the classification of models falls into three main groups: (1) deterministic models and their variants, (2) stochastic models, and (3) special categories that encompass approaches like robust programming, which tackles stochastic problems deterministically. In the following paragraphs, we talk about some notable work conducted on the optimization of fresh food supply chains.

Linear Programming (LP) and Mixed-Integer Programming (MIP) models are often employed in the modeling of fresh produce supply chains. Mansini et al. [6] discussed the unique challenges faced by the fruit industry, including perishability, seasonality, and the need for timely delivery to maintain product quality. The authors provided a model that considers key decision variables such as production levels, inventory levels, and transportation routes. This model aims to find an optimal balance between demand, supply, and distribution while considering constraints and objectives specific to the fruit industry. Overall, their work provides valuable insights into the tactical planning and optimization of supply chains in the fruit industry. However, they fail to consider uncertainties in the yield. Instead, they generated deterministic farms yield values using Monte Carlo simulations.

Other studies have focused on specific aspects of produce supply chain design, such as quality or the identification of optimal locations for hubs or wholesale facilities. For instance, a study by Rong et al. [7] proposed an MILP model that combines food quality decay models with logistics models. Based on the time–temperature profile during storage and transportation of the product, they modeled the quality change of the products throughout the distribution network. Etmandia et al. [8] developed an optimization model to select optimal hub locations and reduce distribution costs.

Rocco and Morabito [9] presented a conceptual framework and a mathematical model to optimize the production and logistics planning in the tomato processing industry. The systematic framework integrates various components, including demand forecasting, production planning, inventory management, transportation, and distribution. Furthermore, the mathematical model quantifies the decision-making processes involved in production and logistics planning while considering multiple objectives, such as minimizing costs, reducing waste, and meeting customer demand. The authors highlight that addressing uncertainties in the parameters used in the solution of their model could add benefits to the result analysis.

Soto-Silva et al. [10] developed a bi-objective optimization model applied to a large-scale apple supply chain that considers various factors such as transportation routes, vehicle capacity, storage facilities, and processing capabilities. The optimization model consists of three mathematical models. The first model seeks to assist in decisions at the purchasing stage where the fresh produce is bought from the producers and transported. The second model permits establishing in which cold chamber to store the purchased produce and its transport [10]. The last one integrates the two above models into a single model using the ϵ-constraint method and provides a solution reducing the overall costs of the supply chain. However, the authors fail to incorporate supply uncertainties from individual producers into their models.

As an alternative to mathematical programming approaches, meta-heuristics have often been developed to solve supply chain design (SCD) problems. Musavi and Bozorgi-Amiri [11] modeled a sustainable hub location–vehicle scheduling problem, in which the transportation fleets at hub nodes are limited in number. To solve it, they employed a non-dominated sorting genetic algorithm-II (NSGA-II) meta-heuristic approach in a multi-objective Mixed-Integer Linear Programming model. Their optimization goals encompassed minimizing transportation costs, preserving food freshness and quality upon delivery, and reducing total carbon emissions to support environmental sustainability objectives. As well as previous works commented on in this article, the authors assumed some of the parameters as deterministic and did not deal with the supply uncertainty. More recent models attempt to simulate possible disruption scenarios in raw material supply to consider supply uncertainty to some extent. This includes the work of Bottani et al. [12], who developed a bi-objective resilient food supply chain design (RFSCD) problem including random values to arbitrarily change the maximum production capacity of every supplier at each iteration. The problem was formulated as a non-linear optimization problem and employs a meta-heuristic solution procedure based on Ant Colony Optimization (ACO) to solve the RFSCD problem. The algorithm generates different resilient designs for the food supply chain as a function of weights set for the objective functions.

Traditional deterministic models using LP or MIP are generally unable to deal with problems that involve uncertainties or give solutions with a high level of risk. The variability of crop yields in agricultural activities is a clear example of uncertainty. Nevertheless, stochastic programming and robust programming (both extensions of linear programming) can help us to address uncertainties, like yield, in the parameters of LP or MIP optimization models for production and logistics planning in food supply chains [2]. For example, Aleotti-Maia et al. [13] proposed a Mixed-Integer Linear Programming (MILP) model for selecting the best post-harvest technology routes. The uncertainty in the environment was modeled as a stochastic programming (SP) problem using a set of market and crop scenarios. They illustrated the application of their model using a case study to find the smallest number of routes to achieve economies of scale and maximize the net profit of a small cooperative of banana producers in Brazil.

Kazaz and Scott [14] evaluated the impact of yield and yield-dependent trading costs on selling prices in a single-period production planning model that maximizes expected return. The model determines the optimal amount of space to lease for production and the number of olives to be sourced from external suppliers, considering uncertainties in yield and yield-dependent costs in a Turkish olive oil supply chain. However, randomness in supply is represented by a stochastic proportional yield (or multiplicative random error term), without considering the impact of weather variability on crop yield. Additionally, transportation costs when acquiring olives from other suppliers were not taken into account.

Several robust optimization (RO)-based frameworks can also be found in agricultural applications to help model uncertainty. Such is the case of Mirzapour Al-e-hashem et al. [15], who addressed a supply chain aggregate production planning problem involving multiple suppliers, multiple manufacturers, and customers. In this work, they presented a Robust Multi-objective Aggregate Production Planning (RMAPP) model that aims to increase revenue by minimizing costs and maximize customer satisfaction by minimizing shortages in all periods. Uncertainty is captured through a set of discrete scenarios represented by probability distributions. In a different work, Bagalian et al. [16] developed a stochastic mathematical formulation for designing network structures for supply chains comprising multiple production facilities, distribution centers, and retailers in markets under supply and demand uncertainty. Demand uncertainty was included in the form of a demand distribution function and the supply uncertainty was modeled in terms of the probability of the occurrence of disruptions in manufacturers, distribution centers, and the connecting links of a supply chain network. The total cost of the chain is the summation of the current investment costs, expected future shortages, and production and transportation costs. The resultant MINLP problem was tackled using robust optimization (RO) and the linearization of the model using a piece-wise linear transformation.

Ahumada et al. [17] presented a stochastic tactical planning model for the production and distribution of fresh agricultural products. Their approach utilized a two-stage stochastic program to evaluate decisions such as timing and crops to plant and labor requirements for the entire season. This would generate different possible scenarios to take into consideration. Then, the second stage calculates the best solution for all the potential scenarios and finds a planting plan that maximizes the expected income for farmers regardless of the outcomes of the random variables such as crop yield and market price.

In a more recent work, Aranguren and Castillo-Villar [18] proposed a bi-objective model, which is solved by applying the ϵ-constraint method and meta-heuristic method to obtain Pareto frontier approximations. The supply uncertainty is tackled using real soil data and climate projections to simulate different crop yield scenarios. Then, a stochastic hub-and-spoke network model is fed with the crop yield scenarios to minimize logistical costs and greenhouse gas emissions. The model is also able to determine the practicality of building consolidation depots to reduce transportation costs.

Apart from simulations, researchers have explored other ways to deal with uncertainty. For example, Baghizadeh et al. [19] proposed an MINLP multi-objective and multi-period model based on a G/M/S/M queuing system to optimize the transportation system and reduce waste in a transportation network. They considered fuzzy uncertainty in several prominent parameters of the problem and solved it using the robust optimization method with the Hybrid Robust Possibilistic Programming (HRPP) approach and the Lagrangian Relaxation (LR) method to solve the model on a large scale.

In summary, the existing literature highlights the extensive use of optimization models, such as LP (Linear Programming) and MIP (Mixed-Integer Programming), in the optimization of fresh food supply chains. Scholars have employed a range of approaches, including deterministic, stochastic, and robust programming, along with meta-heuristics and fuzzy logic, to manage uncertainty and enhance various supply chain components. These aspects include hub locations, transportation routes, and production planning. However, further research is necessary to enhance the effective incorporation of uncertainties and consider additional factors such as weather conditions and yield variability. The findings from our literature review, presented in Table 1, provide a comprehensive summary of the different model types considered and the associated considerations.

### 1.2. Research Gap and Contributions

There is a lack of research on incorporating yield uncertainty specifically in the optimization of strawberry supply chains, which could provide valuable insights for distributors when making informed decisions. The present work introduces a novel model that addresses a significant gap in the literature and makes advancements in the field of fresh food supply chain network design. This model takes into account the imminent variability of crop yield due to climate uncertainty and the associated transportation costs considering refrigerated trailer units. Its primary objective is to design an optimized supply chain network that minimizes the overall cost of meeting the demand for strawberries across multiple customers.

The contributions of this work can be broadly categorized into modeling and application. In terms of modeling, the proposed stochastic formulation incorporates the variability in crop yield per farm by leveraging data from a crop growth and yield simulator rather than relying solely on probability distributions. This approach utilizes real soil attributes of the farms, as well as CO_2_ concentration and climate projections for each farm location over the next twenty-five years. The model’s goal is to minimize investment and transportation costs, taking into consideration various sets of nodes in the network such as farms, consolidation depots, processing plants, warehouses, and customers, along with their respective geospatial locations. From an application standpoint, the model is put to the test through a comprehensive case study based in California, the largest strawberry-growing state in the U.S. This real-world scenario allows for the practical evaluation of the model and provides valuable insights for managing and planning a produce large-scale supply chain.

The structure of this paper follows: Section 2 presents the methodology employed in our design. In Section 3, the proposed model is introduced. Section 4 presents the case study, detailing the data collection process and the development of weather scenarios. Section 5 shows the results of the case study.

## 2. Methodology

**Figure 1:**
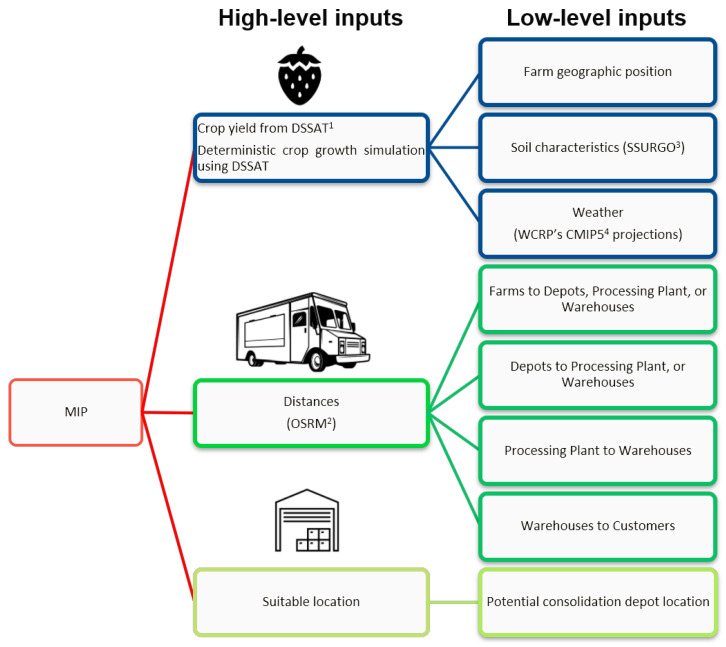
Hybrid simulation-Mixed-Integer Linear Programming (MILP) methodology. ^1^ DSSAT: Decision Support System for Agrotechnology Transfer [21], ^2^ OSRM: Open Source Routing Machine [22], ^3^ SSURGO: Gridded Soil Survey Geographic Database [23], ^4^ WCRP’s-CMIP5: World Climate Research Programme’s Coupled Model Intercomparison Project Phase [24].

**Figure 2:**
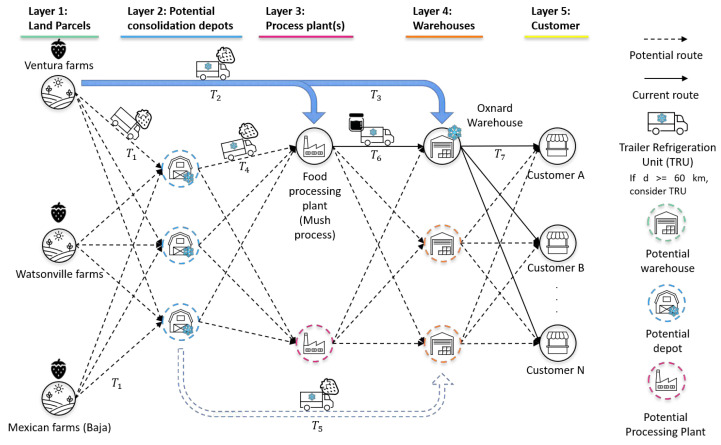
Hub-and-spoke model.

Inspired by the promising preliminary results in [20], the authors significantly extend the study and propose a hybrid methodology to tackle the problem at hand. This methodology consists of two key stages: (1) a simulation, followed by (2) optimization. In the simulation stage, the average crop yield per farm per year is estimated for a desired period, in our case, from 2023 to 2050. This estimation takes into account various factors such as soil properties of the farms, as well as CO_2_ concentration and climate projection inputs, including temperature, solar radiation, and precipitation. The objective of the optimization stage is to identify the optimal design for the CFSC, encompassing aspects like farm selection, depot location, and transportation, with the aim of minimizing the net cost of the strawberry logistics network while satisfying customer demand. Figure 1 and Figure 2 provide a visual representation of the hybrid simulation–MIP (Mixed-Integer Programming) methodology employed in this study, illustrating the potential flows between nodes within the network.

The objective function has two high-level inputs: (1) high-resolution strawberry yield and (2) geospatial parameters. With these inputs, the MIP model determines the optimal flows between nodes and minimizes the net cost of the logistics network while considering the impact of climate change. To test this, a numerical experimentation was conducted to identify the most promising solution procedure by considering a large-scale case study in California, U.S.

## 3. Mathematical Model

The proposed model considers a large-scale CFSC as a hub-and-spoke problem in which we aim to minimize the overall investment and transportation costs of a CFSC, considering several scenarios with different weather conditions and real soil data from the county of Ventura in the state of California. This study focuses on strawberries, as California is one of the leading strawberry-producing states in the United States. The formulation of the new network model consists of five sets of nodes and seven sets of arcs. The first set of nodes represents the farms where strawberry is cultivated. The second set denotes potential sites for depots or hubs, which serve as consolidation points within the supply chain. The third set represents the location of a mush processing plant. The fourth set constitutes centralized warehouses, and the fifth set represents customers’ locations where the strawberries are going to be delivered.

The dynamics of this model consist of trucks delivering strawberries from farms or parcels (P) to either consolidation depots (D), a processing plant, or centralized warehouses (W). When available, strawberries can also flow from depots and the processing plant to warehouses. Then, when a customer requires more product, the strawberries will flow from warehouses to customers (C) to meet the demand. Details regarding the layout of the network are shown in Figure 1. Transportation costs associated with truck usage are associated directly with the distance between the origin and destination geospatial location along with the cost of loading and unloading the unit.

The definitions of all the parameters used in the MIP formulation are as follows:

Sets

- P Set of parcels/farms;
- D Set of potential locations for depots;
- W Set of warehouses;
- C Set of customers;
- $T_{1}$ Set of arcs that connect parcels/farms to the processing plant for all $i\in T_{1}$;
- $T_{2}$ Set of arcs that connect parcels/farms to potential depot locations for all $\left(i,j\right)\in T_{2}$;
- $T_{3}$ Set of arcs that connect parcels/farms to centralized warehouses for all $\left(i,k\right)\in T_{3}$;
- $T_{4}$ Set of arcs that connect potential depot locations to the processing plant for all $j\in T_{4}$;
- $T_{5}$ Set of arcs that connect potential depot locations to centralized warehouses for all $\left(j,k\right)\in T_{5}$;
- $T_{6}$ Set of arcs that connect the processing plant to centralized warehouses for all $k\in T_{6}$;
- $T_{7}$ Set of arcs that connect centralized warehouses to customers for all $\left(k,l\right)\in T_{7}$.

Design variables

- $A^{1}$ Flow along arc $\left(i,1\right)\in T_{1}$;
- $A^{2}$ Flow along arc $\left(i,j\right)\in T_{2}$;
- $A^{3}$ Flow along arc $\left(i,k\right)\in T_{3}$;
- $A^{4}$ Flow along arc $\left(j,1\right)\in T_{4}$;
- $A^{5}$ Flow along arc $\left(j,k\right)\in T_{5}$;
- $A^{6}$ Flow along arc $\left(1,k\right)\in T_{6}$;
- $A^{7}$ Flow along arc $\left(k,l\right)\in T_{7}$;
- $\delta_{j}$ A binary variable that takes the value of 1 if j∈D is used as a depot, otherwise 0.
- $\omega_{k}$ A binary variable that takes the value of 1 if k∈W is used as a warehouse, otherwise 0.

Problem parameters

- $c_{i}^{T_{1}}$ Unit cost per metric ton shipped along $\left(i,1\right)\in T_{1}$;
- $c_{ij}^{T_{2}}$ Unit cost per metric ton shipped along $\left(i,j\right)\in T_{2}$;
- $c_{ik}^{T_{3}}$ Unit cost per metric ton shipped along $\left(i,k\right)\in T_{3}$;
- $c_{j}^{T_{4}}$ Unit cost per metric ton shipped along $\left(j,1\right)\in T_{4}$;
- $c_{jk}^{T_{5}}$ Unit cost per metric ton shipped along $\left(j,k\right)\in T_{5}$;
- $c_{k}^{T_{6}}$ Unit cost per metric ton shipped along $\left(1,k\right)\in T_{6}$;
- $c_{kl}^{T_{7}}$ Unit cost per metric ton shipped along $\left(k,l\right)\in T_{7}$;
- $u_{j}$ Storage capacity of depot j∈D;
- $u_{k}$ Storage capacity of warehouse k∈W;
- $u_{p}$ Storage capacity of the processing plant;
- $\zeta_{j}$ Fixed investment cost to open a depot at node j∈D;
- $\zeta_{k}$ Fixed rental cost to store product in a warehouse at node k∈W;
- $s_{i}$ Supply of strawberries at parcel location i∈P;
- $d_{whole}$ Demand for whole strawberry from each customer location l∈C;
- $d_{mush}$ Demand for strawberry mush from each customer location l∈C;
- $d_{l}$ Total demand for strawberries from each customer location l∈C;
- Losses during the peeling process.

The objective function is the minimization of the total transportation costs, which consist of the logistics operations and infrastructure investment. The first seven terms in (1) denote the summation of transportation costs of the total mass flowing from one node to another one. Each term represents one different set of arcs. The eighth term denotes the investment costs for opening depots. The remaining terms constitute the penalty cost, or rental cost, for storing strawberries in external warehouses. We discuss more about the transportation costs in Section 4.4.

Objective function: minimize transportation cost

(1) $$\begin{array}{c} Min:\sum_{i\in P}c_{i}^{T_{1}}A_{i}^{1}+\sum_{i\in P}\sum_{j\in D}c_{ij}^{T_{2}}A_{ij}^{2}+\sum_{i\in P}\sum_{k\in W}c_{ik}^{T_{3}}A_{ik}^{3}+\sum_{j\in D}c_{j}^{T_{4}}A_{j}^{4}\\ +\sum_{j\in D}\sum_{k\in W}c_{jk}^{T_{5}}A_{jk}^{5}+\sum_{k\in W}c_{k}^{T_{6}}A_{k}^{6}+\sum_{k\in W}\sum_{l\in C}c_{kl}^{T_{7}}\left(\frac{A_{kl}^{7}*wt_{plt}}{Max.wt/plt}+A_{kl}^{7}\right)\\ +\sum_{j\in D}\zeta_{j}\delta_{j}+\sum_{i\in P}\sum_{k\in W}A_{ik}^{3}\zeta_{k}\omega_{k}+\sum_{j\in D}\sum_{k\in W}A_{jk}^{5}\zeta_{k}\omega_{k}+\sum_{k\in W}A_{k}^{6}\zeta_{k}\omega_{k} \end{array}$$

The model is subject to several constraints that govern its operations. First, constraint (2) prevents the total supply of strawberries from parcels to depots, the processing plant, or warehouses from exceeding the available supply. This is crucial because the supply ($s_{i}$) varies across different scenarios, introducing an element of uncertainty. Additionally, constraints (3)–(5) ensure mass conservation for depots, the processing plant, and warehouses, respectively. These constraints guarantee that the amount of strawberries entering each facility matches the amount exiting. Constraint (6) is implemented to meet the demand for whole strawberries. Similarly, constraint (7) ensures that the demand for mushed strawberries is fulfilled. Constraint (8) is employed to satisfy the total demand per customer. Moreover, constraints (9)–(11) impose capacity limits on depots, warehouses, and the processing plant, restricting the amount of strawberries that can be stored at each facility to its maximum capacity. Constraint (12) helps the algorithm set warehouse binary variables ($\omega_{k}$) as zero if a warehouse is not being considered in the optimal solution. Additionally, constraint (13) guarantees that flow is non-negative in any arc. Finally, constraints (14) and (15) are binary constraints that control and indicate the inclusion of depots and warehouses in the optimization process, respectively.

(2) $$\;A_{i}^{1}+\sum_{j\in D}A_{ij}^{2}+\sum_{k\in W}A_{ik}^{3}\leq s_{i}\;\forall i\in P$$

(3) $$\;\sum_{i\in P}A_{ij}^{2}-\left(A_{j}^{4}+\sum_{k\in W}A_{jk}^{5}\right)=0\;\forall j\in D$$

(4) $$\;\sum_{i\in P}A_{i}^{1}+\sum_{j\in D}A_{j}^{4}-\sum_{k\in W}A_{k}^{6}=0$$

(5) $$\;\sum_{i\in P}\sum_{k\in W}A_{ik}^{3}+\sum_{j\in D}\sum_{k\in W}A_{jk}^{5}+\sum_{k\in W}A_{k}^{6}-Loss\geq \sum_{k\in W}\sum_{l\in C}A_{kl}^{7}$$

(6) $$\;\sum_{i\in P}\sum_{k\in W}A_{ik}^{3}+\sum_{j\in D}\sum_{k\in W}A_{jk}^{5}-Loss\geq \sum_{l\in C}d_{l}^{whole}$$

(7) $$\;\sum_{k\in W}A_{k}^{6}-Loss\geq \sum_{l\in C}d_{l}^{mush}$$

(8) $$\;\sum_{k\in W}\sum_{l\in C}A_{kl}^{7}=\sum_{l\in C}\left(d_{l}^{whole}+d_{l}^{mush}\right)$$

(9) $$\;\sum_{i\in P}A_{ij}^{2}-u_{j}\delta_{j}\leq 0\;\forall j\in D$$

(10) $$\;\sum_{i\in P}A_{ik}^{3}+\sum_{j\in D}A_{jk}^{5}+A_{k}^{6}-u_{k}\omega_{k}\leq 0\;\forall k\in W$$

(11) $$\;\sum_{i\in P}A_{i}^{1}+\sum_{j\in D}A_{j}^{4}-u_{p}\leq 0$$

(12) $$\;\sum_{i\in P}A_{ik}^{3}+\sum_{j\in D}A_{jk}^{5}+A_{k}^{6}-\omega_{k}\leq 0\;\forall k\in W$$

(13) $$A^{n}\in R^{+}\;\forall i\in T_{n},\;For\;n=1,2,...,7$$

(14) $$\delta_{j}\in \left\{0,1\right\}\;\forall j\in D$$

(15) $$\omega_{k}\in \left\{0,1\right\}\;\forall k\in W$$

## 4. Case Study

A stochastic cold food supply chain (CFSC) use case is developed based on multiple weather scenarios, utilizing real soil data and climate model inputs specific to Ventura County, California. Strawberry is studied in this work, given that California ranks among the top strawberry-producing states in the United States.

### 4.1. Types of Products

The journey of the strawberry from farms to customers may slightly differ depending on the type of product the customer wants. In this case study, we considered two types of products: (a) a whole strawberry and (b) strawberry mush. The whole strawberry is shipped from farms and gets stored in a centralized warehouse. When the strawberry arrives at the warehouse, it goes through a washing, sorting, and freezing process. Then, it is deposited in a storage freezer. On the other hand, some of the strawberry harvests are first delivered to a processing plant, where they get mushed and put in drums. After that, the drums are driven to the warehouse to be stored in the storage freezer. The transportation of the two types of products will then be from a warehouse to each customer. Customers may ask for only one or both types of products.

### 4.2. Crop Yield Simulations

The estimation of the yield of strawberry is obtained by using the 2017 Decision Support System for Agrotechnology Transfer (DSSAT) software v. 47 [21], which encompasses dynamic crop growth simulation models for a wide range of crops. These crop simulation models enable the simulation of growth, development, and yield based on the interactions between soil, plants, and the atmosphere. They utilize daily weather data, soil information, and detailed crop management practices to generate accurate predictions. For strawberries, the CROPGRO-Strawberry sub-module, developed and refined by Hopf et al. [25], is employed. This model incorporates weather, soil, management, and physiological parameters as inputs to forecast the growth, development, and fruit production of strawberries over time.

In this study, the specific strawberry cultivars and crop management practices implemented in the California farms under investigation were not disclosed due to confidentiality reasons. Therefore, for consistency, the crop management techniques and cultivar (radiance) described in the field experiments conducted by Hopf et al. [25] were assumed. These practices encompassed automatic irrigation, optimal fertilization, and predetermined planting and harvest dates at three- to four-day intervals from 22 November to 15 March. By considering various climate scenarios, multiple crop yields were generated, and all potential outcomes were considered in formulating the overall solution. This introduction of stochasticity into the objective function allowed for a comprehensive assessment of the possible results.

#### 4.2.1. Soil Surface and Profile Information

The importance of the soil surface and its properties in determining crop yield cannot be ignored. As the very foundation on which plants grow and thrive, the condition of the soil surface directly influences the health, productivity, and resilience of agricultural systems. The top layer of soil, known as the topsoil, acts as a vital interface between plants and their environment, playing a crucial role in nutrient availability, water retention, and root development. Furthermore, the diverse physical, chemical, and biological properties of the soil surface interact intricately, shaping the overall fertility and productivity of the land.

The USDA’s Gridded Soil Survey Geographic (gSSURGO) Database [23] is an open-source database that offers soil attribute (properties and characteristics) tables from all the states in the U.S. in the format of an Environmental Systems Research Institute, Inc. (ESRI^®^) file database. We downloaded the soil data for the state of California and processed it using ArcMap [26] v10.8.2 to generate soil rank maps of the counties where farms are located. From these maps, we obtained the necessary soil attributes for estimating yields.

**Figure 3:**
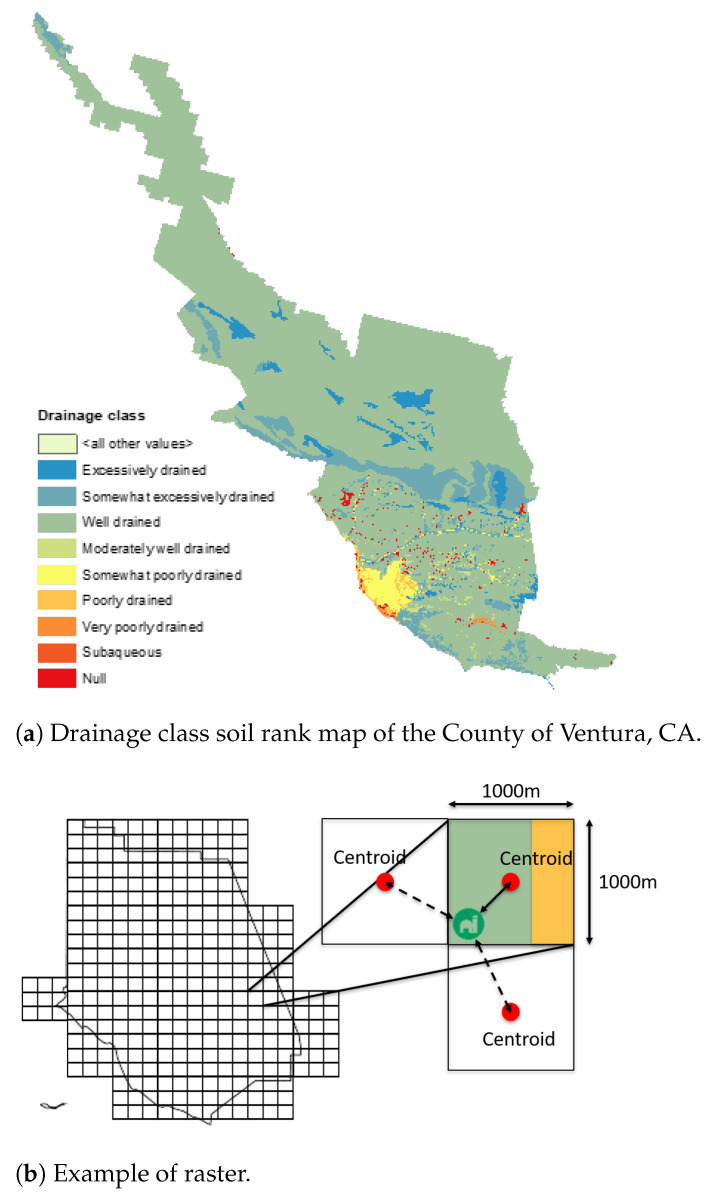
Soil surface and profile information.

Figure 3a demonstrates that counties can exhibit different values of the same attribute. To avoid generalizing the entire area with a single value, we employed a method to divide the soil rank maps into square parcels. While the average size of a farm in the United States is approximately 170 hectares [27], we opted for a grid with cells measuring 100 hectares. By doing so, we aimed to estimate yields at a farm-size level instead of at a county-size level. In cases where a cell displayed multiple attribute values, we assigned the attribute based on the single feature with the largest area within each cell. After generating rasters for each attribute and obtaining the farm locations, we utilized a Python algorithm to calculate the Euclidean distance between each farm and the geospatial centroids of the parcels. This process enabled us to identify the closest centroid and extract the corresponding soil data for each farm. Figure 3b shows an example of this, where the attribute assigned to the cell is the “green” one.

#### 4.2.2. Climate Scenarios

In the proposed mathematical model, the strawberry supply is stochastic, necessitating the generation of multiple future crop yields using different climate scenarios. To obtain these scenarios, we accessed the Downscaled CMIP3 and CMIP5 Climate and Hydrology Projections archive [28]. This archive is a comprehensive collection of global daily climate projections based on the multi-model dataset of the World Climate Research Programme’s [24] Coupled Model Intercomparison Project phase 5 (CMIP5). From this archive, we extracted Localized Constructed Analog (LOCA) time series representing various geographical areas. These time series were derived from 32 different daily models, considering the four Representative Concentration Pathways (RCPs).

For our case study, which spanned from 2023 to 2050, we focused on two future scenarios: RCP 4.5, representing a moderately optimistic scenario, and RCP 8.5, representing the worst-case scenario for greenhouse gas (GHG) emissions. To narrow down our analysis to the relevant areas, we selected the geographical regions where the farms are located. Utilizing a spatial extent selection method with a spatial resolution of 1/16th degree, we precisely defined the desired areas.

Subsequently, we downloaded the LOCA time series from the 32 models, considering all possible combinations of longitudes and latitudes within the selected areas. To determine the closest climate geographic coordinates for each farm, we developed a Python algorithm that calculated the Euclidean distances between the farm locations and the climate geographic coordinates. This step allowed us to extract the corresponding weather projections for each individual farm. Specifically, the parameters used in this study were precipitation, maximum and minimum temperatures, and solar radiation.

**Figure 4:**
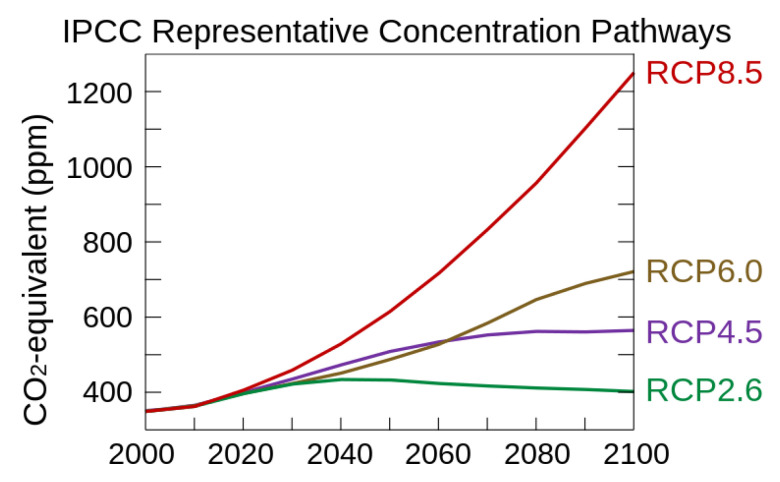
IPCC’s AR5 Representative Concentration Pathways used for projecting climate change to 2100. Graph retrieved from Wikipedia [30].

**Figure 5:**
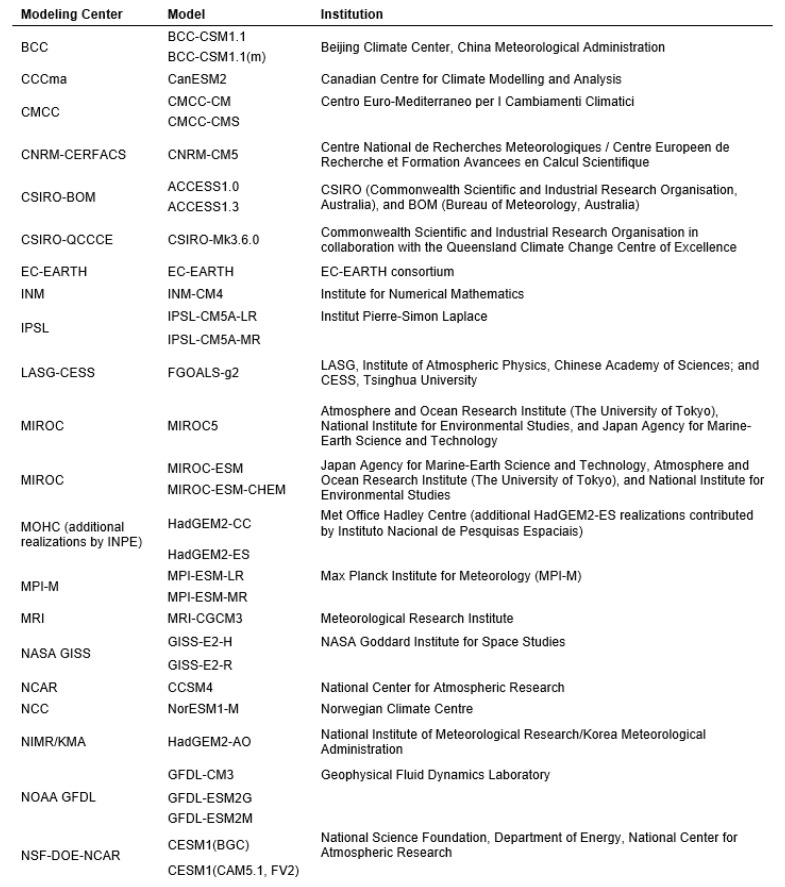
Climate projection models.

To simulate the crop yield, we computed three quartiles (25th, 50th, and 75th percentiles) from the thirty-two time series of each RCP. This process resulted in six different weather scenarios that we utilized in the crop yield simulation. Figure 4 visually presents the estimated greenhouse gas concentration trajectory for each of the four RCPs. For reference, Figure 5 provides a comprehensive list of the models used in the analysis, along with their respective origin institutions. Additionally, we incorporated the yearly CO_2_ concentration projections from the RCP database [29] into the yield simulations.

### 4.3. Geospatial Parameters

The geospatial parameters encompass the geographic location of the different sets of nodes in the network, including farms, depots, the processing plant, warehouses, and customers, along with the corresponding distances between these nodes. The locations of farms, the processing plant, warehouses, and customer locations were provided by our industry partner. Moreover, we tested the introduction of consolidation depots in the supply chain network.

#### Potential Depot Locations

**Figure 6:**
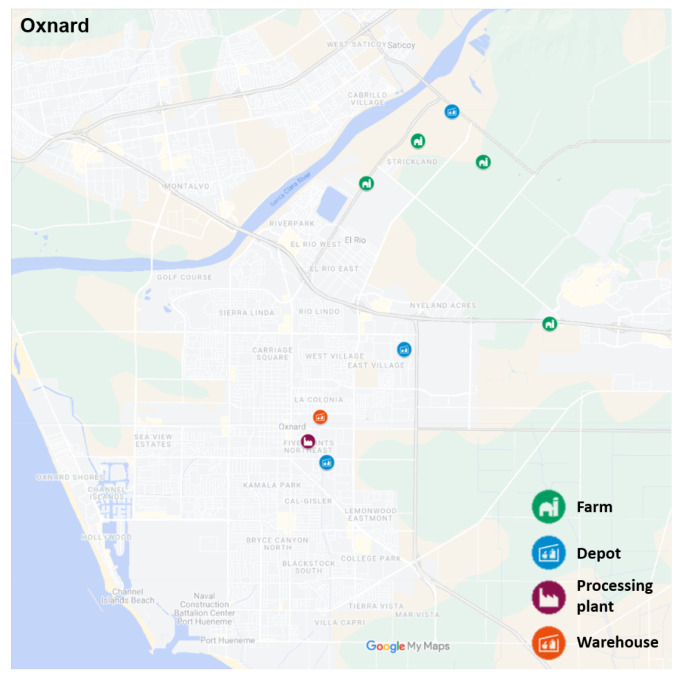
Map of Oxnard showing the location of local farms, the processing plant, and potential depot locations.

The primary objective of introducing new depots into the network is to establish consolidation points for the product, thereby optimizing transportation efficiency before reaching one of the centralized warehouses depicted in layer 4 of Figure 2. For this case study, depots were specifically considered to be strategically located along routes connecting local farms in Oxnard to the processing plant (refer to Figure 6). Additionally, the selection of depot locations required compliance with industrial zoning regulations that permit warehousing activities. To address this requirement, we utilized the Interactive Zoning Map offered by the City of Oxnard [31], an online tool that assisted us in identifying suitable zones with the necessary permits for building storage depots within the city. Based on these considerations, the potential locations identified for this case study involved one depot in close proximity to the farms, another depot positioned between the farms and the processing plant, and a third depot situated adjacent to the processing plant.

In our case study, two depot parameters are particularly relevant: (1) investment costs and (2) storage capacity. Since building a depot involves a significant financial investment, the optimization algorithm plays a crucial role in determining whether incorporating a new depot would be beneficial for the stochastic CFSC. A study conducted by Lamers et al. [32] on the storage and preprocessing of biomass provides valuable insights. According to their findings, the total capital investment (TCI) for opening a depot facility amounts to USD 21,758,808 [32], with an equivalent annual cost (EAC) of USD 3,476,219. These calculations are based on an interest rate (r) of 15% and an estimated project lifetime (t) of 20 years. Furthermore, the preprocessing capacity of conventional pellet processing depots is typically 300,000 metric tons per year [32]. For our case study, we assumed these values and linearly scaled up the capacity to align with the large-scale model proposed in this paper.

### 4.4. Transportation Costs: Costs Along Arcs T

The costs incurred along arcs T encompass both transportation costs per unit and expenses related to loading and unloading the trucks. In this network, there are seven distinct sets of arcs connecting nodes. In order to calculate the total transportation costs for moving strawberries between nodes, we employ the methodology proposed by Aranguren and Castillo-Villar [18]. Their research introduces a formula that incorporates fixed costs and the distance between the origin and destination points, enabling the computation of the transportation cost per metric ton of product. The total cost per arc is determined by the following equation:

(16) $$c^{T}=L+U+\mu +\frac{x\theta \psi }{v\rho^{T}},$$

The parameters used in the transportation cost calculations include the following: loading cost (*L*), unloading cost (*U*), stacking cost (μ), distance (*x*), round trip factor (θ), truck operational cost (ψ), average truck speed (*v*), and truck capacity (ρ).

The first three terms in Equation (16) represent the costs incurred during the loading and unloading of trucks. Meanwhile, the fourth term combines various travel expenses, including operational costs, truck capacity, and the distance *x* between point A and point B for the analyzed arc. To determine the distance *x* associated with each arc, we utilize the shortest route algorithm provided by the Open Source Routing Machine (OSRM) platform [22]. By combining these factors, we can calculate the unitary cost of transporting strawberries through the specific arc. For further details, please refer to Table 2.

In addition, it is not advisable to transport strawberries over long distances using regular trucks. Therefore, when the distance between the origin and destination exceeds a specific threshold, an operational cost adjustment is applied to account for the use of refrigerated trailer units (TRUs) instead of regular trucks. According to Syam et al. [33], regardless of the vehicle type, further data indicated that the average fuel consumption of a typical refrigeration system ranged from 15% to 25% of the vehicle engine’s fuel consumption. For this case study, if the distance *x* is greater than 60 km, the operational cost will be multiplied by a factor of 1.2 to account for the use of TRUs.

## 5. Results and Discussion

To test the optimization model, a comprehensive numerical experimentation was conducted using a large-scale case study in California, United States. The experiments were run on a computer equipped with an Intel(R) Core(TM) i7-11800H processor running at a frequency of 2.30 GHz with 32 GB of random-access memory. Algorithms that were used in this case study to solve the mathematical model were made in Jupyter Notebook 6.5.6 and solved using the Python API of CPLEX 22.1.1 [34] from IBM^®^. The weather data processing was carried out using R Statistical Software [35] v4.2.2.

### 5.1. Numerical Results

The experimentation involved the consideration of 17 farms, 3 consolidation depots, 11 warehouses, and 300 customer locations. Specifically, four farms were located in the Oxnard area, seven farms in Watsonville, CA, and six farms in Mexico. Additionally to the six yield scenarios derived from the climate projections, a tolerance of ±10% for each scenario was taken into account for crop yield variations. In total, there were 18 different scenarios that considered a varying yield for our optimal solution. Our experimentation showed that 18 scenarios can capture the natural variability of the strawberry yield while maintaining a reasonable computational effort and time in attaining an optimal solution. Running a large number of scenarios can substantially increase the computational time required to reach a solution.

The optimal stochastic CFSC model determined that none of the three available depot locations were selected, while all eleven warehouse locations were chosen for all possible yield scenarios. Based on the results, the most favorable scenario in terms of transportation costs to meet the strawberry demand from 300 different locations amounted to USD 1,168,333,199.09. However, there is a possibility of an increase by USD 26,275,187.49. The results for each scenario are presented in Table 3 and Table 4, organized by RCP 4.5 and 8.5, and ordered from the least to the most optimal scenario.

### 5.2. Sensitivity Analysis

To evaluate the impact of weather variability on the supply chain design and strawberry supply, climate change scenarios were tested. The analysis primarily focuses on evaluating the network topology and the availability of strawberry supply to gauge the resilience of the baseline solution under different climate conditions.

The optimization algorithm generated feasible solutions for all 18 scenarios. Notably, scenarios based on the 75th percentile—reflecting higher-than-average rainfall during the planting season—consistently resulted in lower total costs. Specifically, the best-case scenario yielded a cost that was USD 26,275,187.49 lower than the worst-case scenario. The total supply available in the worst-case scenario was significantly lower (56,670.67 mt) compared to the best-case scenario (240,850.36 mt).These results indicate that increased rainfall leads to higher crop yields, enabling demand fulfillment through sourcing from nearby farms, thereby reducing transportation costs. In contrast, scenarios with lower rainfall (25th percentile) resulted in reduced yields, requiring sourcing from more distant farms and thus increasing transportation expenses.

Furthermore, the analysis revealed that out of the 18 possible scenarios, only 3 scenarios would allow the supply chain to meet the demand by sourcing solely from local suppliers. In other words, there is an 83.33% chance that the company will need to seek strawberries from more distant farms. Considering the financial aspect, the logistics company may explore options such as relocating facilities closer to farms that can provide the majority of the supply or exploring alternative shipping methods to mitigate transportation expenses.

**Figure 7:**
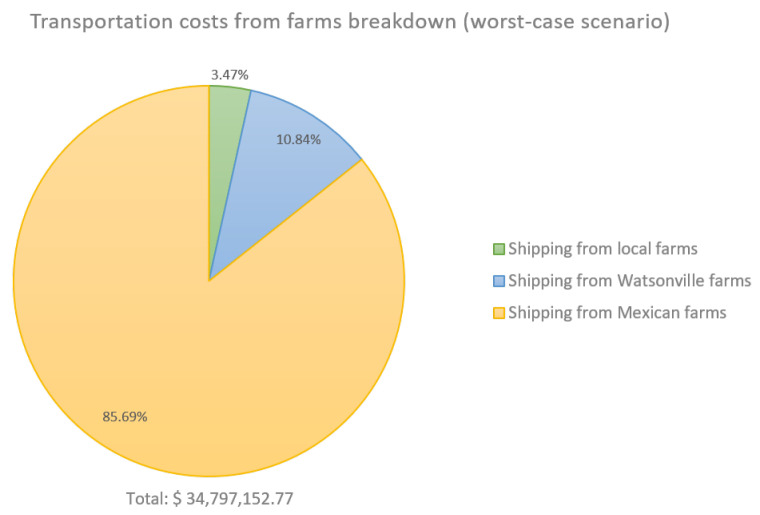
Strawberry supply chain costs breakdown. Worst-case scenario: RCP 4.5/−10%/25th percentile.

**Figure 8:**
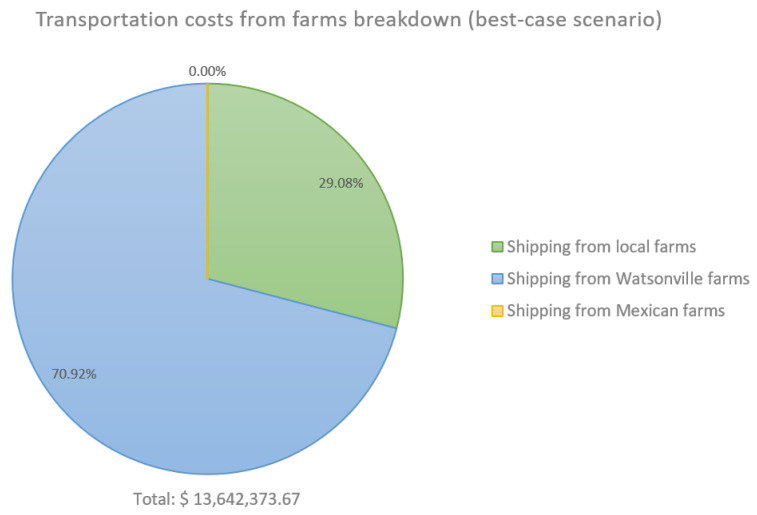
Strawberry supply chain costs breakdown. Best-case scenario: RCP 8.5/+10%/75th percentile.

To provide a more detailed view of the financial impact of climate variability, Figure 7 and Figure 8 present the breakdown of transportation costs from farms to processing plants and warehouses under the least and most favorable scenarios, respectively. In the worst-case scenario, transportation costs from farms totaled USD 34,797,152.77, while the best-case scenario incurred USD 13,642,373.67. As shown in Table 4, the best-case scenario enabled complete demand fulfillment through local sourcing. This translates to a potential increase of USD 21,154,779.10 in transportation costs under unfavorable weather conditions.

Through a comprehensive comparison of the results, the Mixed-Integer Programming (MIP) model has effectively validated the hypothesis of this work: the variability of weather patterns due to climate change can have a substantial effect on strawberry crop yields, thereby disrupting the supply chain and introducing additional transportation costs. This finding underscores the importance of implementing proactive strategies to develop more resilient supply chains capable of adapting to the challenges presented by climate variability, ensuring the continuity of strawberry supply while minimizing costs. By considering climate variability and its impact on crop yield, the optimization model provides valuable insights for strategic planning and investment decisions aimed at enhancing long-term efficiency and robustness.

## 6. Concluding Remarks

### 6.1. Summary of Contributions

The optimization of cold food supply chains (CFSCs), particularly under crop yield fluctuations driven by weather variability, is vital for developing robust and resilient systems. One effective approach to handling yield uncertainty involves simulating crop growth and production and integrating the resulting data into the optimization model. By forecasting farm-level output, it becomes possible to enhance purchasing and transportation decisions with the goal of minimizing costs.

In this study, we propose a hybrid optimization model that integrates a hub-and-spoke structure to address a stochastic CFSC design problem. The case study focuses on strawberry cultivation in California and its distribution across the United States. The model incorporates multi-modal transportation, using conventional trucks for distances under 60 km and trailer refrigerated units (TRUs) for longer hauls. By incorporating climate variability and its influence on yields, the mathematical model aims to reduce transportation costs across various possible scenarios.

Simulation results show that higher rainfall during the growing season, corresponding to upper percentile weather scenarios, leads to increased crop yields, enabling distributors to source strawberries locally and reduce transportation costs. In contrast, lower percentile scenarios characterized by reduced rainfall produce lower yields, compelling distributors to rely on more distant sources, thus incurring higher transportation expenses.

After incorporating the simulated yields into the model, representing all plausible future yield scenarios, the optimization process selects the farms to be used, determines the quantity of strawberries to procure from each, and identifies optimal flow paths across the network to minimize transportation costs while accounting for variable supply. These predictive yield data allow distributors to plan ahead and implement mitigation strategies to reduce recovery time and purchasing costs in the event of supply disruptions. For example, securing contracts with backup suppliers in regions such as Watsonville or Mexico can ensure availability and protect against last-minute, high-cost sourcing.

In summary, the findings highlight that the cold food supply chain is highly vulnerable to climate variability and its impacts on agricultural yield. The proposed stochastic CFSC model is designed to create a resilient and efficient system that can adapt to unforeseen events and disruptions, thereby supporting decision-making for both distributors and consumers. Strategic actions such as securing alternative suppliers, evaluating facility relocation, and optimizing transportation logistics are key to enhancing supply chain resilience.

### 6.2. Practical Implications

The outputs of the optimization model, especially the distribution of strawberry flows, offer actionable insights for fleet utilization and logistics planning. Further, the analysis opens opportunities to assess the viability of relocating processing facilities closer to alternative sourcing regions. The case study reveals that in most scenarios the distributor does not source strawberries from local farms. This insight could prompt an evaluation of opening new processing plants nearer to key production areas, thereby lowering transport costs for processed goods. Currently, the distributor operates eleven warehouses across the United States but none in Mexico. Establishing a consolidation depot or centralized warehouse near Mexican farms could reduce both storage and transport expenses. While the model primarily focuses on transportation costs in a stochastic, cold-stored strawberry supply chain, the proposed hybrid simulation–MIP methodology is flexible and can be extended to consider operational and energy costs. If operational costs in Mexico are lower, locating a new warehouse in that region could be a cost-effective strategy. Noteworthy, the methodology developed in this study is generalizable and can be adapted to optimize cold food supply chains in other countries and for other climate-sensitive perishable crops.

Additionally, the findings of this study have meaningful implications for sustainability planning and logistics policy. By incorporating climate variability into supply chain design, the proposed model has the potential to support the objectives of SDG 12 (Responsible Consumption and Production) by enabling more efficient resource allocation. Additionally, the model contributes to SDG 13 (Climate Action) by promoting anticipatory strategies that reduce the environmental and economic impact of climate-induced disruptions. The use of scenario-based modeling offers a decision support tool for policymakers and industry stakeholders seeking to improve the resilience of perishable food supply chains under future climate uncertainty.

### 6.3. Limitations of the Study and Future Directions

An inherent limitation of this work is that supply chain configurations may vary depending on the choice of climate models or weather prediction sources, highlighting the importance of regularly updating scenario inputs to ensure robust planning. For future work, two main extensions are proposed. First, incorporating storage and in-transit crop losses, along with produce quality tracking, would provide a more complete supply chain assessment. Second, converting the model into a multi-objective framework would allow for the simultaneous minimization of other key factors, such as CO_2_ emissions. These advancements would contribute to a more integrated and sustainable approach to stochastic cold food supply chain optimization.

## Figures and Tables

**Table 1:** Literature review.

				Supply Uncertainty	
References	Methods/Approach	Type	Objective	Probability Dist.	Weather Projections
Masini et al. (2007) [6]	LP ^1^ + MCS ^2^	Deterministic	Single		
Rong et al. (2011) [7]	MILP ^3^	Deterministic	Single		
Etemadnia et al. (2015) [8]	MIP ^4^ + Heuristics	Deterministic	Single		
Rocco and Morabito (2016) [9]	LP ^1^	Deterministic	Single		
Soto-Silva et al. (2017) [10]	MIP ^4^	Deterministic	Single		
Musavi and Bozorgi-Amiri (2017) [11]	MIP ^4^ + Meta-Heuristic (NSGA-II ^5^)	Deterministic	Multiple		
Bottani et al. (2019) [12]	MINLP ^6^ + Meta-Heuristic (ACO ^7^)	Deterministic	Multiple	✓	
Maia et al. (1997) [13]	MIP ^4^	Stochastic	Single	✓	
Kazaz and Webster (2011) [14]	Newsvendor	Stochastic	Single	✓	
Mirzapour Al-E-Hashem et al. (2011) [15]	MILP + RO + LP Metrics	Stochastic	Multiple	✓	
Ahumada et al. (2012) [17]	MIP ^4^	Stochastic	Multiple		
Baghalian et al. (2013) [16]	MINLP ^6^ + RO ^8^ + Piece-wise Linearization	Stochastic	Single	✓	
Aranguren and Castillo-Villar (2022) [18]	Hybrid: Yield Simulations + MILP ^3^ + Meta-Heuristic (SA and PSO)	Stochastic	Multiple		✓
Baghizadeh et al. (2022) [19]	MINLP ^6^ + HRPP ^9^ + LR ^10^	Fuzzy sets	Multiple	✓	
This paper	Hybrid: Yield Simulations + MIP ^4^	Stochastic	Single		✓

^1^ Linear Programming; ^2^ Monte Carlo Simulation; ^3^ Mixed-Integer Linear Programming; ^4^ Mixed-Integer Programming; ^5^ Non-Dominated Sorting Genetic Algorithm-II; ^6^ Mixed-Integer Non-Linear Programming; ^7^ Ant Colony Optimization; ^8^ Robust Optimization; ^9^ Hybrid Robust Possibilistic Programming; ^10^ Lagrangian Relaxation. The ✓ means that the paper included that perspective.

**Table 2:** Transportation costs.

Item	Value ^∗^	Unit
Avg. truck speed (v)	60	Km/h
Truck operational cost (ψ)	48.4	USD/h
Loading cost (L)	3.59	USD/MTon
Unloading cost (U)	3.58	USD/MTon
Stacking cost (μ)	0.44	USD/MTon
Truck capacity (ρ)	21.76	MTon
Round trip factor (θ)	2	-

^∗^ Costs extracted from Aranguren and Castillo-Villar [18].

**Table 3:** Results for RCP 4.5.

Δ Yield	Percentile	Farms Used	Depots	Warehouses	Avg. Supply (mt)	Cost
−10%	25th	17	0	11	56,670.67	USD 1,194,608,386.58
0%	25th	17	0	11	56,670.67	USD 1,190,332,227.52
+10%	25th	16	0	11	60,212.59	USD 1,186,607,165.33
−10%	50th	8	0	11	120,425.18	USD 1,168,407,595.64
0%	50th	6	0	11	160,566.90	USD 1,168,394,944.64
+10%	50th	6	0	11	160,566.90	USD 1,168,388,566.68
−10%	75th	6	0	11	160,566.90	USD 1,168,346,008.80
0%	75th	5	0	11	192,680.29	USD 1,168,336,253.85
+10%	75th	4	0	11	240,850.36	USD 1,168,334,088.73

**Table 4:** Results for RCP 8.5.

Δ Yield	Percentile	Farms Used	Depots	Warehouses	Avg. Supply (mt)	Cost
−10%	25th	16	0	11	60,212.59	USD 1,186,898,725.03
0%	25th	15	0	11	64,226.76	USD 1,182,436,538.05
+10%	25th	14	0	11	68,814.39	USD 1,180,608,885.81
−10%	50th	6	0	11	160,566.90	USD 1,168,394,783.53
0%	50th	6	0	11	160,566.90	USD 1,168,387,679.00
+10%	50th	6	0	11	160,566.90	USD 1,168,380,574.47
−10%	75th	6	0	11	160,566.90	USD 1,168,340,993.58
0%	75th	4	0	11	240,850.36	USD 1,168,335,097.63
+10%	75th	4	0	11	240,850.36	USD 1,168,333,199.09

## Data Availability

The data presented in this study are available on request from the corresponding author. The data are not publicly available due to the confidentiality and privacy of the participants.
